# Patterns and Distribution of HIV among Adult Men and Women in India

**DOI:** 10.1371/journal.pone.0005648

**Published:** 2009-05-21

**Authors:** Jessica M. Perkins, Kashif T. Khan, S. V. Subramanian

**Affiliations:** 1 Department of Health Policy, Harvard University, Cambridge, Massachusetts, United States of America; 2 Department of Population and International Health, Harvard School of Public Health, Boston, Massachusetts, United States of America; 3 Department of Society, Human Development and Health, Harvard School of Public Health, Boston, Massachusetts, United States of America; University of Cape Town, South Africa

## Abstract

**Background:**

While the estimated prevalence of HIV in India experienced a downward revision in 2007, the patterning and distribution of HIV in the population remains unclear. We examined the individual and state-level socioeconomic patterning of individual HIV status among adult men and women in India as well as the patterning of other individual demographic and behavioral determinants of HIV status.

**Methodology/Principal Findings:**

We conducted logistic regression models accounting for the survey design using nationally representative, cross-sectional data on 100,030 women and men from the 2005–2006 India National Family Health survey which, for the first time, provided objective assessments of HIV seroprevalence. Although there was a weak relationship between household wealth and risk of being HIV-positive, there was a clear negative relationship between individual education attainment and risk of being HIV-positive among both men and women. A 1000 Rupee change in the per capita net state domestic product was associated with a 4% and 5% increase in the risk for positive HIV status among men and women, respectively. State-level income inequality was associated with increased risk of HIV for men. Marital status and selected sexual behavior indicators were significant predictors of HIV status among women whereas the age effect was the most dominant predictor of HIV infection among men.

**Conclusions/Significance:**

Although the prevalence of HIV in India is low, the lack of strong wealth patterning in the risk of HIV suggests a more generalized distribution of HIV risk than some of India's high-risk group HIV prevention policies have assumed. The positive association between state economic development and individual risk for HIV is intriguing and requires further scrutiny.

## Introduction

In 2007, UNAIDS and WHO issued a major downward revision of the HIV estimate in India, from 5.7 million (3.4–9.4 million) people in 2005 to 2.5 million (2.0–3.1 million) people in 2006 [1], [2]. Around 0.35% of the population in 2006 was estimated to be HIV infected. The new HIV prevalence estimate represents a combination of data from the recent India National Family Health Survey 3 (NFHS-3) [3], the first national population based survey to include HIV testing, and data from sentinel surveillance among specific populations. The reduction in the HIV estimate was largely attributable to the acquisition of new HIV data from the NFHS-3. Previous national HIV prevalence estimates were derived primarily from sentinel surveillance among pregnant women attending government antenatal clinics and high risk population groups such as sexually transmitted disease clinic attendees, female sex workers, injection drug users, men who have sex with men, migrants, and truckers [3], [4]. The surveillance estimates were likely overestimates of HIV burden as they were not based on representative samples of adults in India [5]. However, they still provided important information because they captured the HIV prevalence among high risk groups who were often missed by population based surveys [1].

The revised HIV estimate prompted many responses to the Indian HIV epidemic including the following: HIV rates seen in the general population in sub-Saharan Africa will not occur in India [6]; the HIV epidemic in India is less generalized than had been thought and that there are greater opportunities to control it [7]; HIV prevention efforts in India should concentrate on high risk groups such as commercial sex workers and their clients, men who have sex with men, mobile populations such as migrant laborers and truckers, people with other sexually transmitted infections, and injection-drug users [6]. These narrow interpretations of the revised HIV prevalence estimate may not, however, improve the effectiveness of HIV prevention efforts because the revised estimate does not necessarily imply a lack of a generalized epidemic.

The salient question for HIV prevention is about the appropriate balance between targeting HIV prevention interventions at high risk groups versus the general population. Therefore, it is important to examine the distribution of HIV prevalence in India, particularly in light of the downward revision. The patterns, distribution, and determinants of HIV throughout the country remain largely undocumented albeit a few local or regional studies [5], [8], [9]. Indeed, HIV affects large numbers of people in India [7]; India ranks third, behind South Africa and Nigeria, in the greatest number of people living with HIV [10]. In this study, we report the patterns and distribution of HIV in India by individual socioeconomic status (SES) and other individual characteristics (demographic and behavioral) as well as the relationship between individual HIV status and state economic indicators.

## Methods

### Data

We analyzed data from the 2005–2006 India National Family Health Survey (NFHS-3), the first national survey to include HIV testing. This survey collected demographic, socioeconomic, and behavioral data, as well as blood samples for HIV testing from nationally representative samples of adult women and men. NFHS-3 was designed to provide a national estimate of HIV in the household population of women aged 15–49 and men aged 15–54, as well as separate HIV estimates for each of the six highest HIV prevalence states – Andhra Pradesh, Karnataka, Maharashtra, Manipur, Nagaland, and Tamil Nadu – and for one low HIV prevalence state – Uttar Pradesh. All women aged 15–49 and all men aged 15–54 living in all sample households in those states were eligible for HIV testing. In the remaining 22 states, HIV testing was conducted in only a sub-sample of six households per enumeration area, and all women aged 15–49 and all men aged 15–54 in those sample households were eligible for HIV testing. Sample sizes in those 22 states were large enough to contribute to a reliable estimate of national HIV prevalence. Blood collection for HIV testing was not conducted in Nagaland due to local opposition, so Nagaland has not been included in the analysis here. In the remaining sample, HIV tests were conducted for 85 percent of the 62,182 eligible women and 78 percent of the 64,175 eligible men in India, resulting in a sample size of 102,946 women and men. Further details on sampling, testing procedures, and basic descriptive statistics can be found in the final report of the NFHS-3 survey [3]. The final sample analyzed in this study consists of 100,030 men and women who provided complete responses for the outcome and all of the covariates (except as noted in the variable description below).

### Outcome

The dependent variable was a binary variable indicating HIV serostatus. Blood spot samples from a finger prick were collected on special filter paper cards for HIV testing. Participation in HIV testing was voluntary; before collecting blood samples for HIV testing, each selected participant was asked to provide informed consent to the testing [11]. Informed consent was obtained separately for the questionnaire interview. HIV testing was conducted in a central laboratory, SRL Ranbaxy in Mumbai, by following a standard testing algorithm designed to maximize the sensitivity and specificity of HIV test results, and an approved quality assurance and quality control plan [12]. The testing algorithm used two HIV enzyme immunosorbent assays, based on different antigens. All discordant samples that were positive on the first test and negative on the second test were retested with the same enzyme immunosorbent assays, and if still discordant, were resolved by Western blot testing. These steps were also prepared for 5–10% of randomly selected samples that tested negative on the first test. For external quality assessment, a subset of dried blood spot samples was retested at an outside reference laboratory using the same algorithm.

### Individual socioeconomic predictors

Socioeconomic measures included household wealth, educational status, employment, caste, and living environment. Wealth was defined in terms of ownership of material possessions by the household [13], with each adult assigned a wealth score based on a combination of 33 different household characteristics that were weighted according to a factor analysis procedure and divided into quintiles [14], [15]. Educational status was measured as level of education attained: no education, primary, secondary, and higher. Caste was based on individual self-identification as belonging to a scheduled caste, scheduled tribe, other backward class, other or no caste group. Scheduled caste consists of castes that are lowest in the traditional Hindu caste hierarchy and, as a consequence, experience intense social and economic exclusion and disadvantage. Scheduled tribes comprise approximately 700 tribes which tend to be geographically isolated with limited economic and social interaction with the rest of the population. Other backward class is a diverse collection of “intermediate” castes that were considered low in the traditional caste hierarchy, but above scheduled castes. An individual's employment status was defined as either unemployed or employed. Living environment was defined as city, town, or rural.

### Individual demographic and behavioral predictors

We included the following demographic characteristics: 5-year age groups and marital status (never married, married or living with a partner, widowed, or separated/divorced) as well as circumcision for men. We also included sexual behavior/knowledge characteristics that are known to increase individuals' HIV susceptibility: lifetime number of partners (3 categories representing 1 partner, 2–4 partners and 5 or more partners), condom use at last sexual contact, previous AIDS test, STD symptoms in the past twelve months, and any knowledge of AIDS. The latter four represent dichotomous variables. Many participants did not provide a response to the questions on circumcision, number of lifetime partners, and condom use at last sexual contact. In order to retain their otherwise valuable information in the analyses, we created ‘Missing’ categories for these three variables.

### State economic predictors

We used the 2005–2006 per capita net state domestic product (PCSDP) (in Indian rupees, INR) [16] and the 2004–2005 Gini coefficient [17] to examine the relationship between state-level socioeconomic status and individual HIV status. The Gini coefficient was only available for 17 states. Therefore, we created a ‘Missing’ category for the other states. We used the 2004–2005 PCSDP for Jammu and Kashmir and Mizoram, as data for 2005–2006 were not available. The Gini coefficient is a measure of income inequality within a state, where 1 indicates extreme inequality and 0 indicates perfect equality. We categorized states into two groups: low Gini and high Gini based on a median split.

### Statistical analysis

We used logistic regression accounting for the survey design (weighting and Primary-Sample-Unit clustering which represented villages or clusters of villages in rural areas and wards in urban areas) to examine the odds of an individual being HIV positive controlling for various demographic, social, and behavioral characteristics, stratified by gender.

### Ethical Review

The 2005–06 National Family Health Survey was conducted under the scientific and administrative supervision of the International Institute for Population Sciences (IIPS), Mumbai, India. The IIPS is a regional center for teaching, training and research in population studies and is associated with the Ministry of Health and Family Welfare, Government of India. The institute conducted an independent ethics review of 2005–06 NFHS protocol. Data collection procedures were also approved by the ORC Macro institutional review board. Informed consent for the survey, the anemia test and the HIV test were obtained by interviewers [18]. The study was reviewed by Harvard School of Public Health Institutional Review Board and was considered as exempt from full review as the study was based on an anonymous public use data set with no identifiable information on the survey participants.

## Results

Table 1 provides HIV prevalence within strata of individual socioeconomic, demographic, and behavioral indicators. Table 2 presents the probability weighted prevalence of HIV positive people per state. HIV prevalence varied across states, from 0.97% in Andhra Pradesh to 0.00% in five states. Table 3 presents the adjusted odds ratios (ORs) and the 95% credible intervals (CIs) for the risk of being HIV-positive associated with individual demographic characteristics, socioeconomic indicators, and behavioral risk factors among men and women, separately, from a single logistic regression model.

**Table 1:** Probability weighted number of participants and HIV prevalence by socioeconomic, demographic, and behavioral indicators from the 2005–2006 India National Family Health Survey 3 sample (n = 100030).

Characteristic	Indicator	Subcategory	Participants	Percent	HIV	Percent
Socioeconomic Status	Education	None	29388	29.51	94	0.32
		Primary	16150	16.22	70	0.43
		Secondary	44418	44.60	101	0.23
		Higher	9634	9.67	10	0.10
	Wealth	Poorest	16505	16.57	40	0.24
		Poorer	18599	18.68	41	0.22
		Middle	20757	20.84	63	0.31
		Richer	21244	21.33	88	0.41
		Richest	22484	22.58	41	0.18
	Employment	Unemployed	39407	39.57	53	0.14
		Employed	60182	60.43	221	0.37
	Caste	Scheduled caste	19258	19.34	54	0.28
		Scheduled tribe	8457	8.49	21	0.25
		Other backward class	40386	40.55	126	0.31
		Other	31489	31.62	74	0.23
	Environment	City	20640	20.72	70	0.34
		Town	13516	13.57	46	0.34
		Rural	65433	65.70	158	0.24
Demographic	Sex	Female	51667	51.88	113	0.22
		Male	47922	48.12	162	0.34
	Age (years)	15–19	18643	18.72	8	0.04
		20–24	16874	16.94	28	0.16
		25–29	15558	15.62	56	0.36
		30–34	13908	13.97	69	0.50
		35–39	12593	12.65	48	0.38
		40–44	10513	10.56	32	0.31
		45–49	8465	8.50	23	0.27
		50–54	3035	3.05	11	0.35
	Marital status	Never married	25917	26.02	27	0.10
		Currently married	70410	70.7	203	0.29
		Widowed	2138	2.15	28	1.30
		Divorced or separated	1124	1.13	17	1.48
	Circumcision	No	42264	42.44	150	0.36
		Yes	4993	5.01	7	0.14
		‘Missing’	52332	52.55	117	0.22
Behavioral	Heard of AIDS	No	27738	27.85	43	0.16
		Yes	71851	72.15	231	0.32
	Condom use at last sex	No	63281	63.54	194	0.31
		Yes	4987	5.01	11	0.23
		Missing	31322	31.45	69	0.22
	STD symptoms	No	98770	99.18	271	0.27
		Yes	819	0.82	3	0.42
	Previous HIV test	No	96197	96.59	242	0.25
		Yes	3393	3.41	33	0.97
	Lifetime Partners	One	67617	67.90	203	0.30
		Two to four	6310	6.34	41	0.65
		Five or more	997	1.00	9	0.95
		‘Missing’	24665	24.77	20	0.08

**Table 2:** Probability weighted sample size per state and frequency of people who are HIV positive from the 2005–2006 India National Family Health Survey.

State	Total Participants		HIV positive	
	*N*	*%*	*N*	*%*
Andhra Pradesh	8167	8.20	80	0.98
Arunachal Pradesh	123	0.12	0	0
Assam	2095	2.10	1	0.07
Bihar	6663	6.69	2	0.03
Chhattisgarh	2219	2.23	1	0.06
Delhi	1368	1.37	2	0.15
Goa	4807	4.83	4	0.09
Gujarat	130	0.13	1	0.59
Haryana	592	0.59	2	0.31
Himachal Pradesh	2046	2.05	2	0.12
Jammu and Kashmir	2659	2.67	4	0.13
Jharkhand	592	0.59	3	0.52
Karnataka	5953	5.98	41	0.68
Kerala	2652	2.66	0	0
Madhya Pradesh	233	0.23	0	0
Maharashtra	9745	9.78	60	0.62
Manipur	196	0.2	0	0
Meghalaya	6544	6.57	15	0.23
Mizoram	101	0.10	0	0
Orissa	3927	3.94	5	0.13
Punjab	2790	2.80	6	0.23
Rajasthan	5405	5.43	4	0.07
Sikkim	71	0.07	1	1.35
Tamil Nadu	6104	6.13	21	0.34
Tripura	415	0.42	0	0
Uttar Pradesh	784	0.79	0	0
Uttaranchal	15426	15.49	12	0.07
West Bengal	7781	7.81	8	0.10
India	99589	100	274	0.28

**Table 3:** Adjusted odds ratios and 95% credible intervals for the association of socioeconomic, demographic, and behavioral indicators with positive HIV status among men (n = 48673) and women (n = 51357).

Characteristic	Indicator	Subcategory	Men OR	(95% CI)	Women OR	(95% CI)
Socioeconomic Status	Education	None	1.89	(1.06, 3.37)	2.44	(1.38, 4.31)
		Primary	1.94	(1.19, 3.15)	2.17	(1.16, 4.06)
		Secondary	1	-	1	
		Higher	0.33	(0.15, 0.71)	0.43	(0.12, 1.52)
	Wealth	Poorest	1.36	(0.55, 3.39)	1.46	(0.64, 3.34)
		Poorer	1.10	(0.50, 2.40)	1.14	(0.53, 2.48)
		Middle	1.30	(0.68, 2.49)	1.37	(0.63, 2.97)
		Richer	1.61	(0.86, 3.00)	1.93	(1.01, 3.69)
		Richest	1	-	1	-
	Employment	Unemployed	1	-	1	-
		Employed	0.81	(0.40, 1.66)	1.68	(0.73 to 1.24)
	Caste	Scheduled caste	0.70	(0.44, 1.11)	0.79	(0.46, 1.36)
		Scheduled tribe	1.05	(0.46, 2.43)	0.55	(0.24, 1.25)
		Other backward class	1	-		-
		Other	0.69	(0.40, 1.18)	0.90	(0.49, 1.65)
	Environment	City	1	-	1	-
		Town	1.46	(0.84, 2.53)	1.43	(0.65, 3.14)
		Rural	1.17	(0.69, 2.00)	1.67	(0.86, 3.23)
	State Economy	PCSDP*	1.04	(1.02, 1.06)	1.05	(1.03, 1.07)
		Low Gini	1	-	1	-
		High Gini	1.67	(1.03, 2.71)	0.84	(0.46, 1.50)
Demographic	Age (years)	15–19	1		1	
		20–24	22.63	(3.47, 147.58)	0.92	(0.31, 2.700)
		25–29	50.32	(7.77, 325.93)	1.29	(0.55, 3.060)
		30–34	62.80	(9.71, 406.27)	1.56	(0.63, 3.860)
		35–39	58.95	(8.61, 403.36)	0.67	(0.23, 1.910)
		40–44	44.99	(6.61, 306.35)	0.46	(0.16, 1.360)
		45–49	36.54	(5.26, 254.01)	0.42	(0.15, 1.170)
		50–54	36.55	(4.53, 294.68)	-	-
	Marital status	Never married	1.08	(0.39, 3.04)	1.89	(0.73, 4.93)
		Currently married	1	-	1	
		Widowed	0.53	(0.11, 2.61)	12.64	(4.97, 32.16)
		Divorced or separated	3.38	(0.94, 12.18)	7.03	(2.58, 19.11)
	Circumcision	No	1	-	-	-
		Yes	0.36	(0.17, 0.77)	-	-
Behavioral Indicator	Heard of AIDS	No	1	-	1	-
		Yes	1.56	(0.70, 3.49)	2.76	(1.58, 4.82)
	Condom use at last sex	No	1	-	1	-
		Yes	0.98	(0.49, 1.97)	0.33	(0.10, 1.03)
		Missing	1.68	(0.74, 3.79)	0.55	(0.23, 1.34)
	STD symptoms	No	1	-	1	-
		Yes	2.48	(0.64, 9.54)	1.41	(0.32, 6.20)
	Previous HIV test	No	1	-	1	-
		Yes	3.84	(2.41, 6.11)	1.52	(0.72, 3.21)
	Lifetime Partners	One	1	-	1	-
		Two to four	1.53	(1.00, 2.32)	2.70	(1.14, 6.41)
		Five or more	1.59	(0.72, 3.51)	36.11	(5.91, 220.48)
		‘Missing’	0.60	(0.22, 1.66)	0.21	(0.10, 0.48)

* Beta coefficient represents for every 1000 Rupee change there is an ‘X’ change in odds.

### Socioeconomic patterning of HIV

Compared to those with secondary education, men with higher education (OR 0.33, 95% CI 0.15–0.71) have a lower odds ratio of being HIV-positive (Table 3). However, those with no education (OR 1.89, 95% CI 1.06–3.37) and those with primary education (OR 1.94, 95% CI 1.19–3.15) have higher odds ratio of being HIV-positive. Women showed a similar pattern; those with higher education were associated with a reduced risk of being HIV-positive whereas women with no education or primary education were associated with increasing risk of being HIV-positive (Table 3). Compared to the richest quintile, the odds ratio of being HIV-positive increased by 61% and 93% for men and women, respectively, in the second richest quintile and by 36% and 46% for men and women in the poorest quintile after controlling for all of the other indicators in the model. These results, however, were not statistically significant.

There were no statistically significant relationships between HIV status and the following indicators regardless of gender: being employed, caste types, and living environments as compared to the reference groups.

### State economic indicators and HIV

For men living in states with high income inequality (as compared to low income inequality), there was an associated 67% increased risk of being HIV-positive (Table 3). There was no effect, however, for women. In contrast, there were similar results for men and women in the relationship between positive HIV status and PCSDP: each 1000 Rupee increase in PCSDP was associated with a 1.04 (95% CI 1.02–1.06) increased odds of being HIV-positive among men and a 1.05 (95% CI 1.03–1.07) increase in the odds for women.

### Demographic and behavioral patterning of HIV

Although men aged 20 years or more were associated with an increased risk of being HIV-positive as compared to 15–19 year old men, women showed no age gradient (Table 3). Marital status was not a strong predictor of HIV status among men as only divorced/separated men appeared to be at greater risk of being HIV-positive though this was not statistically significant. However, being a widowed or divorced/separated woman was associated with statistically significantly higher odds of being HIV-positive (OR 12.64, 95%CI 4.97–32.16 and OR 7.03, 95%CI 2.58–19.11, respectively). Men who were circumcised were statistically significantly less likely to be HIV-positive. Although the associated change in risk probability for the “risky” categories of the sexual behavior/knowledge indicators was in the predicted direction among men, having a previous AIDS test was the only sexual behavior/knowledge indicator that was statistically significantly associated with HIV status (OR 3.84, 95% CI 2.41–6.11), on average in the population after controlling for the other covariates. In contrast, among women who had more than one lifetime partner or had heard of AIDS, the odds of being HIV-positive were statistically significantly higher. Condom use at last time of sex was associated with reduced odds of being HIV-positive among women although this result bordered on statistical significance.

## Discussion

Though India is a very low HIV prevalence country, it has a large number of infected people. On the one hand, the HIV epidemic in India may be more “generalized” than the responses to the revised HIV estimate indicated given the weak social gradient of HIV according to household wealth among both men and women. On the other hand, the epidemic may be focused on socially disadvantaged groups who mainly suffer an educational disadvantage, regardless of gender. The complex nature of these results is similar to other studies. Some studies have found an inverse relationship between socioeconomic status and HIV in wealthy countries [19]–[21]. Other studies have illustrated a strong, positive relationship between socioeconomic status and HIV in sub-Saharan African countries [22], [23]. This study, however, suggests an independent influence of education on HIV status beyond any influence that household wealth may have on individual HIV status. Although India exhibited a relatively inconsistent relationship between household wealth and individual HIV status, the negative relationship between individual education and positive HIV status was stronger for both men and women.

Our results add to the body of mixed evidence on the association between household wealth and HIV [24], [25]. Some argue that poverty creates the conditions – limited access to education, employment, training – for risk-taking and high risk sexual activity, and, thus, increasing exposure to HIV [26], [27]. Others argue that wealth is associated with HIV by enabling individuals to purchase sex and maintain multiple concurrent sexual partnerships which increase exposure to HIV [28]. The varying increased risk for positive HIV status by household wealth in India does not adhere solely to either of these theories. One reason for a weak wealth gradient may be that access to HIV prevention services does not depend on a household's ability to pay, for example, for free condom distribution. Furthermore, although increased wealth may improve access to healthcare facilities which offer services to help reduce HIV transmission, utilization is not guaranteed because agency may not be guaranteed. Thus, solely poverty reducing strategies [27], may not be the most effective intervention to reduce HIV prevalence in India. Similarly, the patterning of HIV status by education shown in this study (reduced risk associated with increased education) is confirmed by research in India [29], and in Africa [30], though contrasted by research in some developing countries [31], [32]. People with greater education may have adopted risk-reduction behaviors more quickly than those with less education because the well educated were more exposed to health promotion messages or more empowered to negotiate protective behaviors with sexual partners [30], [31].

The positive associations that PCSDP and state Gini coefficient, the latter just for men, exhibited with individual odds of being HIV-positive (similar to sub-Saharan Africa [33], [34],) indicate that macroeconomic policy may affect an individual's risk for being HIV positive. Further, among men, the extent of state-level inequality may create conditions under which lower educated men engage in risky sexual behavior whereas women may be less empowered to control their sexual behavior regardless of the inequality of the state in which they live. Perhaps a policy targeting places, in addition to targeting people, would prove successful in addressing HIV in India. Further systematic research is required to explore the variation in HIV prevalence and the specific mechanisms operating at the state level (as well as at the neighborhood level).

The different demographic and behavioral patterning of HIV status between men and women suggests that groups differing on factors apart from just SES will be at high risk. Future studies should explore why age is important for men and not for women, and why multiple sexual indicators are predictive of a woman's HIV status, but not for men. These different patterns provide support for a generalized approach to HIV prevention so that all of the various groups at increased risk are likely to be reached by prevention programs. At the same time, if prevention interventions are targeted at a specific gender, taking into account age, marital status, and behavioral indicators may increase effectiveness because it may allow reaching out to certain groups of people beyond the typical “high risk” groups. Given these mixed results, India may want to be cautious in pursuing a solely “high risk” group strategy (referred to in the responses to the revised estimate) because it may not be the most effective way to combat HIV in India. However, the inverse relationship between education and HIV status should not be ignored as there is a clear relationship of increased risk for those with less education, presenting a different type of “high risk” group.

There are several limitations to this study due to the cross-sectional nature of the data, which does not allow examination of causal effects and transitional phenomena. First, we cannot establish whether SES causes HIV or HIV leads to low SES, the latter which has been well-established [35]. Second, for many HIV-positive adults, the infection may have preceded their sexual and other behaviors recorded in the survey. The unclear order of events may have biased some of the associations. The strength and direction of the relationship between SES and HIV prevalence and the roles of risk behaviors and protective factors are likely to change over time, depending on the stage and spread of the epidemic [36]. Third, wealthier HIV positive individuals are likely to survive longer than poorer HIV positive individuals because of better nutrition and access to health care. With cross-sectional data (and the lack of information on antiretroviral care), we could not account for selective survival of wealthier respondents. Finally, this analysis was based on face-to-face self-reported behaviors, which may have led to misreporting of particularly sensitive items, like sexual behavior. When data on sexual behavior were collected in Bagalkot District using a polling booth survey methodology [37], which collects sensitive information in an anonymous fashion, reported levels of risk behavior were much higher. There is evidence that women tend to underreport and men tend to exaggerate their sexual activity [38]–[40]. Furthermore, the degree of misreporting may be different across wealth and education quintiles.

Although the rate of HIV infection in India differs from Africa's HIV trajectory, the epidemic continues to demand a serious and sustained national commitment [41]. The lack of a clear social gradient of HIV according to wealth may indicate a “generalized” epidemic in India. However, the evidence of an educational gradient implies that the lower educated represent a high risk group for targeted prevention efforts. Further, this study has highlighted several types of high risk groups that represent people beyond those who are traditionally thought of as “high risk”, evidence which may be interpreted as supporting a picture of a more generalized epidemic than originally thought. Although the Indian Government's response to the country's HIV epidemic reflects a sincere, intensive, and long-term commitment to effective HIV prevention and care [4], [42], prevention efforts which ignore some evidence of a “generalized” epidemic of HIV, or ignore other types of “high risk” groups, may prove inadequate, at best, for national AIDS control policy in India.
